# Association between Spouse/Child Separation and Migration-Related Stress among a Random Sample of Rural-to-Urban Migrants in Wuhan, China

**DOI:** 10.1371/journal.pone.0154252

**Published:** 2016-04-28

**Authors:** Yan Guo, Xinguang Chen, Jie Gong, Fang Li, Chaoyang Zhu, Yaqiong Yan, Liang Wang

**Affiliations:** 1 Wuhan Centers for Disease Prevention and Control, Wuhan, China; 2 Department of Epidemiology, University of Florida, Gainesville, Florida, United States of America; 3 Pediatric Prevention Research Center, Wayne State University, Detroit, Michigan, United States of America; Temple University School of Medicine, UNITED STATES

## Abstract

**Background:**

Millions of people move from rural areas to urban areas in China to pursue new opportunities while leaving their spouses and children at rural homes. Little is known about the impact of migration-related separation on mental health of these rural migrants in urban China.

**Methods:**

Survey data from a random sample of rural-to-urban migrants (n = 1113, aged 18–45) from Wuhan were analyzed. The Domestic Migration Stress Questionnaire (DMSQ), an instrument with four subconstructs, was used to measure migration-related stress. The relationship between spouse/child separation and stress was assessed using survey estimation methods to account for the multi-level sampling design.

**Results:**

16.46% of couples were separated from their spouses (spouse-separation only), 25.81% of parents were separated from their children (child separation only). Among the participants who married and had children, 5.97% were separated from both their spouses and children (double separation). Spouse-separation only and double separation did not scored significantly higher on DMSQ than those with no separation. Compared to parents without child separation, parents with child separation scored significantly higher on DMSQ (mean score = 2.88, 95% CI: [2.81, 2.95] vs. 2.60 [2.53, 2.67], p < .05). Stratified analysis by separation type and by gender indicated that the association was stronger for child-separation only and for female participants.

**Conclusion:**

Child-separation is an important source of migration-related stress, and the effect is particularly strong for migrant women. Public policies and intervention programs should consider these factors to encourage and facilitate the co-migration of parents with their children to mitigate migration-related stress.

## 1. Introduction

### 1.1 Rural-to-urban migration and family separation

Domestic migration is increasingly common in many developing countries across the globe [1, 2]. Every year, a large number of rural residents in China migrate to cities to earn money and to look for better opportunities [3]. Data from the 2014 China Statistical Yearbook indicated that the number of rural-to-urban migrants reached as many as 245 million [4], more than the total population in many countries in the world.

When moving from a rural area to a city in China, rural migrants cannot change their legal residential status from rural to urban because of the restriction of *Hukou policy*. *Hukou* is a household registration system that was established by the Chinese government after 1958 [5]. Anyone who do not hold a urban *Hukou* cannot gain access to many governmental resources allocated for urban residents, including opportunities for employment, housing, social welfare, healthcare, and child education [6, 7]. For example, without an urban *Hukou*, all migrants from rural area cannot receive the same medical insurance offered to urban residents. Their medical insurance is warranted through their Rural *Hukou* and can only be accessible from the institutions in rural homes even if they are working and living in the city. Child education cost in urban is also much higher in the urban area than in the rural area [8]. As a result of the *Hukou* system, many rural residents migrate to cities alone, leaving spouses and children in rural homes so that they can access all the social welfare, health, and education services designated for them [9, 10]. Data from the 2012 Monitoring Report of China's Migrant Population showed that among rural residents who migrate, 39.34% migrate alone [11]. A thorough investigation between family separation and migration stress is highly needed to promote mental health of the millions of rural migrants in China.

### 1.2 The Concept of migration-related stress

In theory, stress occurs when the impact of a stressor is greater than an individual’s capability to cope with [11, 12]. Using this definition, migration-related stress occurs if stressors related to migration are greater than migrants’ capability to cope. Life events (such as get married, divorce, and death of a relative) are commonly used in assessing stress in general [13–15]. An inspection of these events shows that few of them are directly related to rural-urban migration in China. Therefore, live events cannot be used to assess migration related stress. In a recent research, Chen et al used four components to describe domestic migration-related stress, including separation from people in the place of origin, rejection by people in the destination, lack of self-confidence, and maladaptation to life in the destination [16]. A measurement instrument, the Domestic Migration Stress Questionnaire (DMSQ) is thus established and available for use to assess domestic migration-related stress [17, 18].

### 1.3 Spouse separation and migration stress

Beside the restriction by *Hukou*, low income is significant barrier that prevents migrants from bringing their families together, as incomes earned from the available jobs are often inadequate for renting or buying properties large enough for a whole family [19]. The lack of adequate education prevent many rural migrants to take high paying jobs; what left for them are jobs with low pay and less attractive to urban residents, such as handling corpses, cleaning sewage and chemical waste, engaging in construction, and working as handyman [20]. As an options, many migrants let their spouses stay rural homes to take care of young children and old parents [14]. It is our expectation that spouse separation will increase the likelihood for married migrants to experience greater stress compared to non-married migrants[21]. Previous studies have shown that long-term spouse separation significantly reduces migrants’ physiological and emotional satisfaction [22, 23]. Some married migrants who separate with their spouse choose to cohabitate with other migrants to form “temporary couples” as a coping strategy [24]. However, little is known spouse separation increases stress among rural migrants in China.

### 1.4 Child separation and migration stress

Relative to spouse separation, child separation could be more stressful. As described in the previous sections, in addition to spouse separation, the *Huhou* system, education level and the economic conditions may also prohibit many rural migrants from bringing their young children with them when migrating to city. The number of ‘‘left-behind children” (LBC) with either or both parents working in city is increasing [25]. Currently there are approximately 61 million of LBC living in rural China [26]. Findings from published studies indicate that LBC are more likely to suffer from mental health, behavioral and nutritional problems, compared to the non-LBC counterparts [27, 28]. Therefore, migrant parents in city with LBC at rural home may be at extra high risk to experience migration-related stress. Positive relationships between parent separation and child stress are reported in the literature [29, 30]. But few studies, if any, investigate the relationship between child separation and migration-related stress among migrant parents who work and live in urban China.

### 1.5 Women and migration stress

Data from multiple sources indicate that 42–45% of rural-to-urban migrants in China are female [4, 31]. More research is needed to assess migration stress among these female migrants[32]. Relative to males, female migrants are more likely to work in labor-intensive and service sectors because of the increased opportunities in these sectors, including hotels, restaurants, retails, and tourisms [33, 34]. Therefore, migration-related stress for females may differ from that for their male counterparts. Furthermore, female migrants may be more sensitive than males to child and spouse separation. Understanding gender differences in migration-related stress is of great significance to promote social, behavioral and mental wellbeing of the rural migrant women and their children; however, to the best of our knowledge, no reported study has investigated this issue.

### 1.6 Purpose of this Study

The purpose of this study is to investigate migration-related stress, particularly the relationship between spouse and child separation and migration-related stress. Previous studies on rural-to-urban migrants focused primarily on general stress and its impact on health [35–37], and the mental health issues of LBC [38, 39]. In this study, we focused on migration related stress and its relationship with child and spouse separation. Our goal is to provide data for evidence-based intervention programming and decision-making to reduce stress and to enhance parental and child health for millions of rural-to-urban migrants in China.

## 2. Methods and Materials

### 2.1 Ethics statement

The Institutional Review Boards’ approval of the study protocol was obtained from Wuhan Center for Disease Prevention and Control, Wuhan, China, the Wayne State University, Detroit, and the University of Florida, Gainesville, USA. All participants provided written consent before participating in the survey.

### 2.2Participants and Sampling

The participants of this study were recruited in Wuhan, the capital city of Hubei Province. Wuhan has a total population of 10 million and per capita GDP of $12,708 [40]. Rural-to-urban migrant in this study was defined as possessing a legal rural *Hukou*, 18–45 years old, working or living in city for at least one month by time of the survey day. Participants were selected using a GIS/GPS-assisted stratified random sampling design. First, residential areas in urban Wuhan were divided, on computer, into mutually exclusive geounits of 100 meters by 100 meters as the primary sampling frame (PSF). Altogether 60 geounits were randomly selected from the PSF. Considering cost-effectiveness of the study, relatively more geounits were sampled in districts with a larger number of migrants using the *optimal design method*[41]. Second, Approximately 20 households were then random sampled from each geounit. Last, one person per household per gender was selected from the sampled households and recruited to participate in the study. To ensure independence, one participant by gender in one household was selected using the random digits method. To ensure adequate sample size, 20% extra geounits were added, considering of absence and refusal.

A total of 1414 rural migrants were invited to participate, and 1293 (91.44%) agreed to participate and completed the survey. To evaluate the reliability of the responses, a self-assessment question at the end of the questionnaire was asked, “To what extent do you think your responses to all the survey questions are reliable?” (1 = totally reliable; 2 = up to 80% are reliable, 3 = about half are reliable, 4 = about 20% are reliable, and 5 = totally unreliable). Participants with an answer of 3 or above (n = 158) were excluded. In addition, participants who reported being divorced or widowed (n = 19) or with missing data regarding child or spouse separation (n = 3) were excluded, yielding a final sample of 1113 migrants for analysis.

The commercial software ArcGIS, version 10.0 (ESRI, Inc., Redlands, CA) was used for geounit sampling. The GPS receiver (Garmin Oregon 450, Garmin, Ltd) was used to assist researchers in locating the sampled geounits and to assess the actual area size of a geounit for calculating sample weights. The GIS/GPS-assisted stratified random sampling was detailed elsewhere [18, 42].

### 2.3 Procedures of data collection

Field survey was completed between March 2011 and December 2013. A small team of core investigators from Wuhan Center for Disease Prevention and Control (Wuhan CDC) first went to the field with assistance of a GPS receiver to locate a sampled geounit and households located with the geounit. A plan for field survey of the geounit was then established with the assistance of local community health center(s) and street committee (s) to whom the sampled geounit belonged. On the pre-scheduled date, a team of trained data collectors (typically 4 to 5 researchers from Wuhan CDC, plus a number of public health graduate students) went to the site to administer the survey.

Data were collected using the Migrant Health and Behavior Questionnaire [43], delivered with Audio Computer-Assisted Self Interviewing (ACASI) techniques. The survey was completed in a private room in the participant’s home or a community health center. One trained data collector was nearby for assistance if needed. Other persons were asked to leave during the survey.

### 2.4 Variables and their measurements

#### Spouse separation (SS)

Spouse separation was defined as participants who were either married or have a fiancé and whose spouse does not live with him/her in Wuhan. Marital status was determined using the questions “What is your marital status (answer options: unmarried, married, divorced or widowed, and others)?” For those who indicated that they were not married, they were further questioned: “Do you have (answer options: yes, no).” Participants who were unmarried and had not a steady boyfriend or girlfriend were defined single. Participants who reported being married or had a steady boyfriend/girlfriend were further questioned, “Does your spouse/fiancé live with you in Wuhan [the current city] (yes/no)?” Participants that responded in the negative to this question were coded as having spouse separation, otherwise no spouse separation (NSS).

#### Child separation (CS)

Child separation was defined as participants who were married and had children and whose children do not live together with them in Wuhan. Two items were asked as “How many children do you have” and “How many of them are with you in Wuhan [current city]”. Participants were coded as having child separation if the reported number in Wuhan (second item) was less than the total number of children (first item). Otherwise no child separation (NCS) was coded.

With defined CS and SS, child separation only (living with spouse with at least on child left at rural home), spouse separation only (living with children with only spouse left in rural home), and double separations (with spouse and at least one child left in rural homes) were defined for further analysis.

#### Migration stress

Migration stress was assessed using the DMSQ. This instrument was developed and tested among rural-to-urban migrants in China and showed adequate reliability and validity (Cronbach alpha = 0.93) [16]. The DMSQ is a 16-item scale with four subconstructs of Separation (SE), Rejection (RE), Lack of Self-Confidence (SC), and Maladaptation (MA). Each subcontruct consists of four items. A typical item for SE is: “I worry so much about my family members and relatives who remain so far away from me in my hometown”; a typical item for RE is: “I have been rejected or stared at by others in the city merely because of my appearance or accent”; a typical item for SC is: “I would never get rich no matter how hard I tried”; and a typical item for MA is: “I did not expect that it would be so difficult to make money here [in the city]”. Individual item was measured using a 5-point Likert rating scale with 1 = “*Never*” and 5 = “A*lways*”. Cronbach alpha was 0.95 for our data. Mean scores were computed for DMSQ and its four subconstructs (range from 0 to 5) for analysis such that higher scores indicating greater migration-related stress.

#### Demographic variables

Demographic variables in this study included age (in years), marital status (married, not married), total number of children, education (middle school or less, high school or higher), and monthly income (Chinese currency RMB with 1 dollar = 6.2 RMB Yuan, <1000, 1000–2000, >2000). Some of them were used as covariates in multivariate modeling analysis.

### 2.5 Statistical analysis

The standard descriptive statistics (mean, standard deviation, and rate, proportion, and 95% CI) were used to characterize the study sample. The survey estimation method for the multi-level random sampling design [44] was used for data analysis, including the survey means to estimate and compare DMSQ scores across various comparison groups. In the comparison analysis, a non-inclusive 95% CI of an estimate was used as evidence of significant differences at p<0.05 level. The effects of spouse separation only and child separation only were analyzed separately first, followed by an in-depth analysis of the interaction of the two on migration-related stress. Stratified analysis by gender was also conducted to assess male-female differences. All statistical analyses were performed using the SAS version 9.1.3 (SAS Institute, Cary, NC).

## 3. Results

### 3.1 Characteristics of study sample

In Table 1, among 1113 participants, 50.58% were male, 79.69% were married, 32.70% had an education level of high school graduate or above, and 38.55% had a monthly income greater than 2000 Chinese Yuan. Among those who reported being married, 94.36% had one or more children. Among those who were single, 37.61% reported having a fiancé and no one reported having children. Among those who were married or had a steady boy/girlfriend, 16.46% reported separation from spouse/fiancé. Among the participants who had children, 25.81% reported child separation; and among the participants who were married and had children, 5.97% reported both spouse and child separation (double separation).

**Table 1:** Basic Characteristics of the study sample.

Variables	Male	Female	Total
Total, *n (%)*	563 (50.58)	550 (49.42)	1113 (100.00)
Age (years)			
18–24	126 (22.38)	101 (18.36)	227 (20.39)
25–34	201 (35.70)	205 (37.27)	406 (36.48)
35–45	236 (41.92)	244 (44.37)	480 (43.13)
Mean (*SD*)	32.14 (8.16)	32.79 (7.59)	32.46 (7.89)
Education attainment, *n (%)*			
Middle school or less	357 (63.41)	392 (71.27)	749 (67.30)
High school or higher	206 (36.58)	158 (28.73)	364 (32.70)
Monthly income (RMB), *n (%)*			
<1000yuan	58 (10.30)	162 (29.45)	220 (19.76)
1000~2000yuan	215 (38.19)	249 (45.27)	464 (41.69)
>2000yuan	290(51.51)	139 (25.28)	429 (38.55)
Marital status, *n (%)*			
Single	152 (27.00)	74 (13.45)	226 (20.31)
Married	411 (73.00)	476 (86.55)	887 (79.69)
Single			
No	102 (67.11)	39 (52.70)	141 (62.39)
Yes	50 (32.89)	35 (47.30)	85 (37.61)
Persons with spouse or boy/girlfriend, *n (%)*			
No	102 (18.12)	39 (7.09)	141 (12.66)
Yes	461 (81.88)	511 (92.91)	972 (87.34)
Number of children, *n (%)*			
0	29 (7.06)	21 (4.41)	50 (5.64)
1	218 (57.07)	284 (62.42)	502 (59.98)
2	148 (38.74)	154 (33.85)	302 (36.08)
3+	16 (4.19)	17 (3.74)	33 (3.94)
Number of children living at current city, *n (%)*			
0	112 (29.32)	67 (14.73)	179 (21.39)
1	176 (46.07)	276 (60.66)	452 (54.00)
2	83 (21.37)	96 (21.10)	179 (21.39)
3+	11 (2.88)	16 (3.52)	27 (3.23)
Child separation^#^, *n (%)*			
No	263 (68.85)	358 (78.68)	621 (74.19)
Yes	119 (31.15)	97 (21.32)	216 (25.81)
Spouse separation*^#^, *n (%)*			
No	357 (77.44)	455 (89.04)	812 (83.54)
Yes	104 (22.56)^#^	56 (10.96)	160 (16.46)
Both spouse/child separation^#^, n(%)			
No	344(90.05)	443(97.36)	787(94.03)
Yes	38(9.95)	12(2.64)	50(5.97)

*; Including singles who were separated from their boy/girlfriend.

^#^; The difference was significant between male and female, *P*<0.05.

### 3.2. Spouse separation and migration stress

Data in Table 2 indicate that the mean DMSQ scores, overall and by sub-constructs range from 2.43 to 3.11 for the married, and 1.98 to 2.46 for the singles. Participants who were married scored significantly higher than the singles in overall scale and subconstructs of Separation (SE), Rejection (RE) and Maladaptation (MA). Among those who were married/had fiancé, separation was negatively associated with RE, MA and LS.

**Table 2:** Differences in DMSQ score between single, married with/without spouse separation, by gender [Mean (95%CI)].

DMSQ/ Subconstructs	Single (141)	Married or having boy/girlfriend		
		Total (972)	NSS (812)	SS (160)
**Total sample (*N = 1113*)**				
Lack self-confidence (LS)	2.24 [2.00, 2.49]	2.70[2.65,2.75]	2.72 [2.66, 2.78]	2.59 [2.46, 2.72]
Separation (SE)	2.27 [1.96, 2.57]	2.58[2.53,2.64]	2.60 [2.54, 2.66]	2.50 [2.36, 2.65]
Rejection (RE)	2.46 [2.20, 2.72]	3.11[3.05,3.17]	3.13 [3.06, 3.20]	3.00 [2.86, 3.15]*
Maladaptation (MA)	1.98 [1.77, 2.19]	2.43[2.36,2.50]	2.44 [2.37, 2.52]	2.35 [2.21, 2.48]*
Lack self-confidence (LS)	2.26 [1.97, 2.55]	2.67[2.61,2.74]	2.71 [2.64, 2.78]	2.51 [2.35, 2.67]*
**Male (*n = 563*)**				
Overall	2.28 [2.04, 2.53]	2.68[2.62,2.75]	2.69 [2.62, 2.76]	2.66 [2.50, 2.80]
Lack self-confidence	2.32 [2.05, 2.60]	2.57[2.50,2.64]	2.57 [2.50, 2.65]	2.55 [2.37, 2.73]
Separation	2.47 [2.14, 2.80]	3.06[2.99,3.13]	3.08 [3.00, 3.16]	3.01 [2.83, 3.18]
Rejection	2.03 [1.80, 2.26]	2.49[2.42,2.57]	2.49 [2.40, 2.58]	2.49 [2.33, 2.66]
Maladaptation	2.31 [2.07, 2.56]	2.62[2.53,2.71]	2.63 [2.53, 2.72]	2.58 [2.37, 2.79]
**Female (*n = 550*)**				
Overall	2.16 [1.64, 2.68]	2.71[2.64,2.79]	2.75 [2.67, 2.83]	2.44 [2.27, 2.62]
Lack self-confidence	2.16 [1.47, 2.85]	2.60[2.53,2.67]	2.62 [2.55, 2.69]	2.40 [2.15, 2.66]
Separation	2.45 [2.04, 2.86]	3.15[3.06,3.25]	3.18 [3.08, 3.27]	2.99 [2.73, 3.24]
Rejection	1.88 [1.50, 2.26]	2.36[2.26,2.47]	2.40 [2.29, 2.52]	2.03 [1.85, 2.21]*
Maladaptation	2.16 [1.47, 2.85]	2.73[2.64,2.83]	2.78 [2.68, 2.89]	2.35 [2.15, 2.55]*

*p < 0.05

NSS: no spouse separation; SS: spouse separation.

Further analysis by gender found that the female married migrants with spouse separation (SS) scored significantly higher than those with no-spouse separation (NSS) in overall DMSQ, and subcontracts RE and MA. No significant differences were found among males.

### 3.3 Child separation and migration stress

As shown in Table 3, participants with child separation (CS) scored significantly higher on the DMSQ (2.88 [2.81, 2.95] vs. 2.60 [2.53, 2.67]) as well as the subconstructs SE, RE, and MA, compared with those with no child separation (NCS).

**Table 3:** Difference in Scores of DMSQ between the participants with or without child separation among the married and having children, by gender [Mean (95%CI)].

DMSQ/ Subconstructs	Total (837)	NCS (621)	CS (216)
**Total sample (*N = 837*)**			
Overall	2.71[2.65, 2.76]	2.60[2.53, 2.67]	2.88[2.81, 2.95]*
Lack self-confidence (LS)	2.59[2.53, 2.65]	2.53[2.46, 2.60]	2.66[2.60, 2.72]
Separation (SE)	3.13[3.06, 3.19]	2.98[2.91, 3.06]	3.32[3.23, 3.41]*
Rejection (RE)	2.42[2.35, 2.49]	2.35[2.27, 2.43]	2.53[2.42, 2.65]
Maladaptation (MA)	2.69[2.62, 2.76]	2.54[2.46, 2.61]	3.00[2.87, 3.13]*
**Male (n = 382)**			
Overall	2.71[2.63, 2.79]	2.63[2.55, 2.70]	2.73[2.59, 2.88]
Lack self-confidence	2.59[2.50, 2.67]	2.52[2.43, 2.60]	2.66[2.54, 2.79]
Separation	3.09[3.01, 3.18]	3.02[2.93, 3.11]	2.94[2.83, 3.05]
Rejection	2.51[2.42, 2.60]	2.41[2.34, 2.49]	2.59[2.40, 2.78]
Maladaptation	2.65[2.54, 2.76]	2.55[2.46, 2.64]	2.74[2.54, 2.94]
**Female (n = 455)**			
Overall	2.70[2.63, 2.78]	2.57[2.46, 2.67]	2.99[2.90, 3.08]*
Lack self-confidence	2.59[2.52, 2.66]	2.55[2.44, 2.65]	2.65[2.59, 2.71]
Separation	3.16[3.06, 3.26]	2.93[2.82, 3.04]	3.61[3.48,3. 73]*
Rejection	2.34[2.24, 2.44]	2.28[2.14, 2.41]	2.49[2.35, 2.63]
Maladaptation	2.72[2.63, 2.81]	2.52[2.39, 2.64]	3.20[3.00, 3.40]*

*p < 0.05

NCS: no child separation; CS: child separation.

Both male and female participants with child separation (CS) scored slightly higher in the DMSQ and its subconstructs than those with no-child separation (NCS). However, the difference was statistically significant only for females on total DMSQ and its two subconstructs SE and MA.

Compared with male with CS, the females with CS scored significantly higher in the DMSQ (2.99 [2.90, 3.08] vs. 2.73[2.59, 2.88], *p* < .05) and two subconstructs SE (3.61 [3.48, 3.73] vs. 2.94[2.83, 3.05], *p* < .05) and MA (3.20 [3.00, 3.40] vs. 2.74[2.54, 2.94], *p* < .05).

### 3.4 Interaction effects of spouse and child separation in migration stress

Table 4 depicts the interaction effects of spouse separation (SS) and child separation (CS). The participants with spouse and child were divided into four comparison groups including no any separation (NSS + NCS), child separation only (NSS+CS), spouse separation only (SS+NCS) and double separation (SS+CS). Generally the participants with child separation only (NSS + CS) scored highest in the overall DMSQ and its subconstructs, and those with spouse separation only (SS+NCS) scored the lowest. Significant differences were also observed between NSS + CS and other comparison groups in the overall DMSQ scores and its two subconstructs SE and MA (*p* < .05 for all).

**Table 4:** Interaction effects of spouse-child separation in scores of DMSQ, by gender [Mean (95%CI)].

DMSQ/ Subconstructs	NSS		SS	
	NCS	CS	NCS	CS
**Total sample (*N = 837*)**				
Overall	2.66[2.58,2.73]	2.91[2.84,2.99]*	2.55[2.41,2.70]	2.71[2.47,2.95]
Lack self-confidence (LS)	2.58[2.51,2.65]	2.67[2.60,2.74]	2.47[2.29,2.65]	2.61[2.47,2.75]
Separation (SE)	3.05[2.97,3.13]	3.36[3.28,3.44]*	2.96[2.79,3.13]	3.13[2.84,3.42]
Rejection (RE)	2.40[2.31,2.50]	2.57[2.45,2.69]	2.34[2.20,2.48]	2.37[2.01,2.74]
Maladaptation (MA)	2.59[2.51,2.68]	3.06[2.91,3.20]*	2.44[2.26,2.63]	2.72[2.41,3.02]
**Male (n = 382)**				
Overall	2.70[2.62,2.77]	2.68[2.53,2.84]	2.60[2.43,2.77]	2.97[2.62,3.32]
Lack self-confidence	2.55[2.46,2.64]	2.65[2.51,2.79]	2.52[2.32,2.72]	2.74[2.52,2.97]
Separation	3.14[3.04,3.24]	2.89[2.78,3.00]	2.97[2.78,3.17]	3.20[2.87,3.52]
Rejection	2.48[2.39,2.58]	2.52[2.32,2.73]	2.42[2.26,2.58]	2.90[2.42,3.37]
Maladaptation	2.61[2.51,2.72]	2.68[2.46,2.89]	2.50[2.27,2.72]	3.05[2.59,3.51]
**Female (n = 455)**				
Overall	2.62[2.51,2.73]	3.08[2.99,3.17]*	2.41[2.15,2.66]	2.49[2.27,2.71]
Lack self-confidence	2.60[2.51,2.70]	2.68[2.61,2.75]	2.33[1.94,2.73]	2.51[2.32,2.69]
Separation	2.98[2.86,3.09]	3.71[3.61,3.80]*	2.93[2.60,3.25]	3.08[2.64,3.52]
Rejection	2.33[2.18,2.48]	2.60[2.45,2.74]	2.09[1.86,2.33]	1.94[1.66,2.21]
Maladaptation	2.57[2.45,2.70]	3.34[3.09,3.59]*	2.29[2.01,2.56]	2.44[2.16,2.71]

*p < 0.05

NSS: no spouse separation; SS: spouse separation; NCS: no child separation; CS: child separation.

Among females, participants with child separation *only* (NSS + CS) scored significantly higher on DMSQ (3.08[2.99, 3.17]) and its two subconstructs SE (3.71[3.61, 3.80]) and MA (3.34[3.09, 3.59]), when compared to the other three groups. And they also scored significantly higher on RE (2.60 [2.45, 2.74]) when compared to SS + NCS (2.09 [1.86, 2.33]) and SS+CS (. 1.94 [1.66, 2.21]).

Compared with the male, the female participants with children separation only (NSS + CS) scored significantly higher on the DMSQ overall and its two subconstructs SE and MA.

## 4. Discussion and Conclusions

In this study, we investigated the association between family separation (including child separation and spouse separation) and migration stress using data collected from a large random sample of rural-to-urban migrants. Findings of our study demonstrate that relative to different types of family separation, child separation is the most significant stressor for rural-to-urban migrants in China; furthermore this relationship is more pronounced among women than men. We also noted that migration stress was more serious for married migrants than for singles. To the best of our knowledge, this is the first study to report data on migration-related stress and its relationship with family separation with data collected from a large random sample. These findings are of great significance to inform policy decision-making and public health intervention to improve mental health of millions of rural-to-urban migrants in China.

### 4.1 Married with spouse separation associated with higher level of migration stress

Our results indicated that married migrants experienced higher levels of migration-related stress than singles. Relative to those who are married, rural migrants who are single may be in some advantages to quickly adapt to and blend in the new environment. The legal and economic conditions prevented many rural migrants from bring their family members to city with them [14]. This could have caused many married migrants to experience greater migration-related stress compared to their single counterparts [21]. Rural migrants living with spouse in city are also associated with increased migration stress. We believe that this seeming contradictory finding can be explained by the fact that many of migrants living with spouse in city also left their children at home, 19.83% (166/837) in this survey. Among the participants migrated with spouse, the scores of DMSQ, SE and MA were all much higher if with child separation. To migrants, if their children are left with wife or husband, they may worry little and have more courage to overcome their difficulties at a new city.

### 4.2 Child separation is associated with the greatest level of migration stress

Compared with other types of family separation, child separation is by far the most significant predictor of migration stress. Participants with child separation, regardless of living with a spouse or living alone, scored higher on the DMSQ than those without child separation. Compared with rural migrants in general [34], the mean scores of DMSQ and the four subconstructs were higher among migrants with child separation. Among the four DMSQ subconstracts, perceived separation (SE), rejection (RE) and maladaptation (MA) to urban life are more important than self-confidence (SC) in predicting the migration-related stress.

The significance of child separation for migration-related stress among rural migrants could be due to the nature of parent-child relationship. The obvious rural-urban distance and the long-term separation may have prevented migrant parents from fostering a solid parent-child bonding and attachment relationship. In addition to affecting their LBC, this distant separation will increase parental stress [45]. In addition, many other factors may have also contributed to the increased migration stress, such as parental awareness of the large rural-urban differences in child cares, lifestyle, and education; the Hukou-related barriers preventing parents from bringing their children with them to city, [27, 46]. Additional research is needed to investigate these mechanisms supporting the development of effective prevention programs and interventions.

### 4.3 Lack of significant effect of child and spouse double separation

Contrary to our expectation, the level of migration stress is not significantly higher for migrants with experience double separations, i.e., separation from both spouses and children, compared to those with spouse separation only, child separation only, or no separation at all. This result was verified with multivariate regression models controlling for covariates (results are available upon request). This seemingly unexpected result may be reasonable. First, migrants who are separated from both spouse and child are verily likely to have one spouse staying at rural home and taking care children, reducing the stress for those who migrate away in city. Second, in our study we observed that single rural migrants ((migration alone) are associated with the lowest level of migration-related stress. Migration without bringing children for the married, similar to migration alone for singles, may facilitate rural migrants to settle down in city, to find a job and to adapt to the new urban environment and lifestyle. Additional research is needed to further test these hypotheses.

### 4.4 Female migrants experience greater stress from child separation

Our study shows that the impact of child separation affects male and female migrants differently. The association between child separation and migration stress is always stronger for females than for males no matter if the data are analysis for the whole sample or stratified by types of separation. In addition, female migrants living with spouses but separated from children exhibited the highest level of stress. Findings from our analysis also suggest that child separation put migrant mothers at increased stress by increasing their feelings of being separated from families and hometowns, being rejected by urban residents, and difficulty adapting to urban life. Our findings of the significance of child separation on mothers’ stress are consistent with reported studies by others that mothers are more likely to be stressed than fathers after they move to a city with children left behind in rural areas [47, 48].

There are limitations to this study. First, data used for this analysis were collected through a cross-sectional survey. Therefore, no causal conclusion is warranted without longitudinal data to rule out any potential reverse impact of stress on spouse/child separation. Secondly, participants in this study were selected from one city. Caution is needed when generalizing the findings of this study to other places within and outside of China. Lastly, data on duration of separation was not collected, preventing us from further assessing potential dose-response relationship between separation and migration stress.

Despite these limitations, this study is the first to investigate the relationship between child/spouse separation and domestic migration, using the newly established DMSQ instrument for stress assessment. Findings of this study provide new data supporting the need for research to develop effective interventions and health policies to protect millions of rural migrants and their children, particularly migrant mothers. For example, programs assisting family reunion may be an effective intervention to reduce maternal stress. Policy changes are also implied to amend the *Hukou* system and ensure the same rights and opportunities for rural migrants working and living in urban areas.
